# Candidate genes related to growth and milk production in three Anatolian goats revealed by GWAS

**DOI:** 10.1007/s00335-026-10203-w

**Published:** 2026-02-02

**Authors:** Eymen Demir, Umit Bilginer, Huriye Doğru, Burak Karacaören, Hasan Meydan, Zeynep Çiftçi, Serdar Yağci, Sarp Kaya, Taki Karsli

**Affiliations:** 1 Department of Animal Science, Faculty of Agriculture, Akdeniz University, 07070 Antalya, Türkiye; 2 Department of Animal Science, Michigan State University, East Lansing, MI 48824 USA; 3 Department of Medical Services and Techniques, Vocational School of Burdur Health Services, Burdur Mehmet Akif Ersoy University, 15100 Burdur, Türkiye; 4 Department of Agricultural Biotechnology, Faculty of Agriculture, Akdeniz University, 07070 Antalya, Türkiye; 5 Department of Biology, Faculty of Science, Hacettepe University, 06800 Ankara, Türkiye; 6 Ministry of Agriculture and Forestry, General Directorate of Agricultural Research and Policies, 06800 Çankaya, Ankara Türkiye; 7 Department of Animal Science, Faculty of Agriculture, Eskişehir Osmangazi University, 26160 Eskişehir, Türkiye

## Abstract

This study aimed to identify candidate genes related to birth weight (BW), live weight at 90th day (90-LW), and lactation milk yield (LMY) in Hair (HAI), Honamlı (HNM), and Kabakulak (KBK) goats, providing a genome-wide basis for further selection strategies. The genome-wide efficient mixed model association approach was assessed with 309.342 bi-allelic single nucleotide polymorphisms (SNPs) across 481 animals to detect significant variants and their corresponding genomic regions. False discovery rate (FDR) correction and suggestive significance threshold were applied to identify SNPs having direct and potential effects on traits of interest, respectively. A total of 138 outlier SNPs (20 in HNM, 47 in HAI, and 71 in KBK) exceeded suggestive significance, of which 12 SNPs were identified to meet Benjamini-Hochberg’s FDR criterion, suggesting that the overlapped protein-coding genes directly influence the phenotypic traits of interest. This approach revealed that the *ID4* and *CXCR4* genes directly affect BW and LMY traits in HNM goats, whereas no significant associations were observed regarding these traits in HAI and KBK goats. Additionally, a total of 8 protein-coding genes were identified to directly influence the 90-LW trait in HAI (*MFSD1*, *CHMP4C*, and *MAP1B*) and KBK (*IGSF21*, *RALY*, *ZNF507*, *SLC38A10*, and *PGBD5*) goats. The remaining 126 suggestive SNPs, on the other hand, either overlapped or were located near 103 protein-coding genes, indicating their potential effects on growth and milk yield. Both direct and potential protein-coding genes identified in this study seem promising for designing comprehensive selection strategies to improve growth and milk traits in Anatolian goats.

**Supplementary Information:**

The online version contains supplementary material available at 10.1007/s00335-026-10203-w.

## Introduction

Having occurred approximately 12,000 YBP, the domestication process of animals and plants has dramatically changed the lifestyle of humankind. Via domestication, ancient society adopted a sedentary lifestyle, accompanied by substantial transformations in the food chain (Vigne 2011; Larson and Fuller 2014). Since its domestication nearly 11.000 YBP in the Fertile Crescent, the domestic goat (*Capra hircus*) has played a vital role in the economy and culture of various human communities (Zeder and Hesse 2000). Even today, goats are reared to produce economically important traits (such as milk, meat, hides, and fiber) and continue to represent a key source of livelihood in rural areas (Vahidi et al. 2014; Karslı and Demir 2024). Compared to other livestock species, goats possess several outstanding advantages, including their ability to adapt to marginal and harsh environmental conditions and utilize low-quality pastures (Boyazoglu et al. 2005; Sejian et al. 2021).

Türkiye contributes to the world’s animal genetic resources by homing to five registered goat breeds known as Hair (HAI), Honamlı (HNM), Kilis, Angora, and Norduz (Daskiran et al. 2018). Besides, various unregistered subvarieties distinct from these breeds in terms of yield and morphological traits are distributed across different geographical zones of Anatolia (Aslan et al. 2022; Demir 2024). For example, Pavga, Çandır, and Kabakulak (KBK) have been reported as subvarieties of the HAI breed (Yilmaz et al. 2012; Karsli et al. 2020; Bilginer et al. 2025). In Türkiye, goat farming is the most practiced around the Taurus Mountains, which extend parallel to the Mediterranean coast, starting from the Teke region in southern Anatolia (between the provinces of Antalya, Burdur, and Isparta) (Daskiran et al. 2018). Within this region, Antalya province is characterized by the intensive breeding of the HAI and HNM goats, while the KBK is reared in a restricted geographic area between Antalya and Muğla provinces (Karsli et al. 2020; Aslan et al. 2022). HAI has a unique adaptation ability against diverse climatic conditions since it has been reared across all geographic regions of the country for centuries. On the contrary, HNM and KBK are reared in restricted geographic zones with lower population sizes, which puts them at some future risks, such as inbreeding depression, genetic bottlenecks, and extinction (Yilmaz et al. 2012). HNM is registered as a distinct breed due to having specific morphological and behavioral patterns. Indeed, several studies based on the comparison of native Turkish goat breeds have confirmed that HNM is morphologically distinguishable from other breeds in terms of arched nose phenotype (Akbaş and Saatci 2016), while their grazing behaviours are different than those of the HAI breed (Akbaş et al. 2017). KBK, on the other hand, remains a subvariety of the HAI breed, whereas several studies confirmed its distinctiveness regarding genetic and morphological basis (Yilmaz et al. 2012). In Türkiye, grassland-based production systems have been mainly adopted to reduce the feeding cost, while there has been a lack of comprehensive selection strategies to improve meat and milk traits, forcing farmers to rear native goat breeds for dual purposes (milk and meat). However, over the past century, several meat-type, milk-type, and dual-purpose goat breeds have been developed worldwide via traditional selection studies, which rely on pedigree and phenotypic records (Groeneveld et al. 2010). Thanks to the traditional selection studies, the breeding value of animals has been estimated by using diverse statistical approaches to increase genetic gain from one generation to another in numerous livestock species (Goddard et al. 2010). It is noteworthy that the collection of phenotypic data regarding economically important traits not only requires intensive labor but is also a time-consuming and costly process in livestock production. Moreover, the low heritability of quantitative traits hinders the achievement of the desired level of genetic gain (Zhang et al. 2011). Fortunately, this limitation has been overcome through the implementation of genomic selection (GS) by providing researchers and breeders with new opportunities in livestock improvement.

The first step of GS studies is to identify candidate genes and genomic regions related to traits of interest in the reference population. Over the past 30 years, traditional marker methods based on polymerase chain reaction (PCR) have been utilized to identify genomic regions associated with economically important traits (quantitative trait loci or QTLs) in goats, as in other livestock species (Cano et al. 2007; Abadi et al. 2009; De La Chevrotiere et al. 2012). However, PCR-based marker techniques allow for the analysis of only a limited number of loci, making them insufficient for uncovering the genetic basis of quantitative traits controlled by polygenic inheritance. As a result, the success of selection has remained below the desired levels. On the other hand, rapid advances in SNP array and whole-genome sequencing technologies, along with progress in bioinformatics, have enabled the production of high-density single nucleotide polymorphism (SNP) data from livestock genomes. These advancements have made genome-wide association studies (GWAS) a popular approach to associate genetic variations with phenotypic records to identify genomic regions significantly affecting the quality and quantity of economically important traits (Schmid and Bennewitz 2017; Saravanan et al. 2020). Recent studies, either based on array technologies or whole genome sequencing technologies, have revealed numerous candidate genes related to growth (*MAPK3*,* LDB2*,* LRP1B*,* COL14A1*,* ZNF148*,* TTC39C*, etc.) and milk (*MPP7*, *PRPF6*, *DNAJC5*, *TPD52L2*, *HNF4G*, *LAMA3*, *FAM13A*, *EPHA5*,* METTL*,* SLC1A 8*,* PHACTR1*,* FMO2*,* ECI1*,* PGP*,* ABCA3*,* AMDHD2*, etc.) traits in different goat breeds (Zhang et al. 2021; Selionova et al. 2024; Shangguan et al. 2024; Zhao et al. 2025). As articulated by Bilginer et al. (2022), SNP arrays are the most commonly preferred genotyping techniques for genetic studies since they are time-efficient, reliable, accurate, and easy to apply, while they have some disadvantages. Indeed, arrays are developed based on some reference genomes, hindering to capture of unique SNPs in local livestock breeds (Geibel et al. 2021). These kinds of SNPs could be detected by whole genome sequencing technologies, while they are still unaffordable for smallholder breeders (Doublet et al. 2024). In this regard, next-generation sequencing technologies such as double-digest restriction-site associated DNA sequencing (ddRADseq) remain powerful tools to retrieve genome-wide genetic data, including novel and breed-specific variations, in a cost-efficient manner. Currently, genomic regions identified via GWAS are incorporated into GS programs to further improve the accuracy and efficiency of genetic gain in livestock species.

Despite their economic and cultural value, the genetic architecture of key traits in Anatolian goat breeds remains largely unexplored, primarily due to the scarcity of comprehensive studies. A considerable number of previous studies, on the other hand, utilized traditional molecular marker techniques based on PCR to monitor polymorphisms (Demir et al. 2020; Karsli et al. 2020; Aktaş et al. 2024). Accordingly, comprehensive genome-wide studies employing high-density SNP data are essential for elucidating the genetic architecture of economically important traits and guiding the development of efficient selection strategies. In this regard, this study is the first attempt to evaluate high-density SNP data obtained through ddRADseq from the reduced representation of the whole genome of HAI, HNM, and KBK goats using a GWAS approach to identify candidate genes associated with growth and milk yield.

## Materials and methods

### Sampling strategy and recording phenotypic data

Before the experiment, the herds belonging to the “Breeding Under Farmer Conditions (BUFC)” project operated by the General Directorate of Agricultural Research and Policies, Ministry of Agriculture and Forestry, were visited in Elmalı, Korkuteli, and Kaş districts of Antalya to sample unrelated and phenotypically distinguishable animals. 384 animals belonging to HAI (180 females and 20 males) and HNM (164 females and 20 males) were sampled from both Elmalı and Korkuteli districts, while 192 samples of KBK (172 females and 20 males) were chosen from herds located in Kaş and the western part of Elmalı district. Detailed information about inbreeding coefficients, population stratification, and geographic locations of the sampling strategy for the studied Anatolian goats was previously given in Bilginer et al. (2025).

Data on birth weight (BW) and live weight at 90th day (90-LW) of sampled animals were retrieved from the BUFC database, while lactation milk yield (LMY) was estimated by the Trapezium II method as recommended by the International Committee for Animal Recording (ICAR). A previous study reported that Kilis goats reared in Anatolia may reach higher LMY in the third lactation relative to the second and fourth lactations (Yılmaz Tilki and Keskin 2021), coinciding with the stage at which udder morphology is considered to reach functional stability. Therefore, we included only animals in their third lactation to minimize age-related effects and obtain more accurate LMY estimates. Milk yield was recorded at five different stages during lactation. The lactation period of HAI, HNM, and KBK goats raised in the province of Antalya ranges from 150 to 180 days. Milk yield on the first control day was measured within the first 10 days after parturition. The second control day measurement was taken between the 40th and 50th days of lactation, corresponding to the onset of the peak yield period. The third control day measurement was conducted between the 85th and 95th days, which coincides with the weaning period. Milk yield on the fourth control day was recorded between the 130th and 140th days of lactation, and the final control day measurement was taken between the 160th and 170th days, representing the late lactation period. These measurements were further subjected to the equation given below to estimate LMY per animal:

$$\mathrm{X} = [(\mathrm{k}_{1}\;\mathrm{A}) + ((\mathrm{k}_{1}+ \mathrm{k}_{2})/2) \mathrm{a}_{1} + \cdots+ ((\mathrm{k}_{\rm n} -1 + \mathrm{k}_{n})/2)\mathrm{a}_{\rm n}+ (\mathrm{k}_{n}\; \mathrm{C})],$$

where k_1_ and k_2_ represent milk yield at the first and last control day, respectively, a and n define the range and number of the control, respectively, A stands for the interval between birth and first control, and C is the interval between the last control and dry period. Phenotypic differences among breeds were evaluated using the Kruskal-Wallis test, as data violated assumptions of normality (Shapiro-Wilk test) and homogeneity of variances (Bartlett’s test). Post-hoc pairwise comparisons were performed using Dunn’s test with Bonferroni correction for multiple testing.

### Molecular experiments and SNP calling

The detailed information regarding molecular experiments, including DNA extraction, ddRADseq-based genomic library preparation, and sequencing, as well as the processing of raw genetic data, was provided in Bilginer et al. (2025). Briefly, 0.5 ml blood samples were subjected to the GeneJET Genomic DNA Purification Kit (Thermo K0722) to extract genomic DNA. The success of DNA extraction was confirmed by the 1% agarose gel electrophoresis, while the Qubit 4™ Fluorometer (Thermo Fisher Scientific) was employed for DNA quantification. Extracted DNA samples were used to prepare genomic libraries according to the ddRADseq technique (Peterson et al. 2012) with minor modifications. Unlike the standard 96 samples procedure, a total of 12 genomic libraries, each covering 48 samples, were prepared to increase coverage depth. Genomic libraries were sequenced via the Illumina HiSeq X Ten instrument with paired-end option (2 × 150 bp) to retrieve raw reads. The BCFTools (Danecek et al. 2021) pipeline was used to obtain SNP data belonging to only autosomal chromosomes. At the final stage, the standard PLINK (Chang et al. 2015) filtration (–maf 0.05, –geno 0.1, –mind 0.1, and –hwe 1e−6) was performed to obtain SNPs with a high call rate. In this study, a total of 481 animals genotyped with 309.342 bi-allelic SNPs remained across HAI (*n* = 156), HNM (*n* = 168), and KBK (*n* = 157) goats to perform GWAS analysis.

### GWAS analysis and gene annotation

GWAS analyses were conducted using GEMMA v0.98.1 (Zhou and Stephens 2012) and R v4.0.4 (https://www.r-project.org) software. Analyses were performed separately for each breed (HAI, HNM, KBK) via the following univariate linear mixed model fitted for each trait:

$$\:y=W\alpha\:+x\beta\:+u+\epsilon\:$$

where $\:y$ is an n-vector of phenotypic observations (BW, 90-LW, and LMY); $\:W$ is the design matrix for fixed effects; $\:\alpha\:$ is the vector of fixed effect coefficients (herd, birth year, age of dam, intercept, and sex); $\:x$ is the vector of SNP genotypes; $\:\beta\:$ represents the allele substitution effect; u is the vector of random polygenic effects with u ~ N(0, λτ^−1^K), where τ⁻¹ is the variance parameter, λ is the ratio of polygenic genetic variance to residual variance and K is the centered genomic relationship matrix constructed from genome-wide SNPs following VanRaden (2008); ε is the vector of residual errors with ε ~ N(0, τ⁻¹I_n_), where I_n_ is an n × n identity matrix.

In this model, SNP markers, herd, birth year, age of dam, intercept, and sex were included as fixed effects, whereas the kinship matrix was included as a random effect to account for relatedness among individuals. Model selection was performed by comparing alternative models using the Akaike Information Criterion (AIC) and genomic inflation factor (λ). The mixed linear model showed the lowest AIC and reduced genomic inflation compared with the general linear model, indicating a better fit and lower risk of false positives. Therefore, the mixed linear model with fixed and random effects was chosen as the optimal model for GWAS analysis.

Population structure and relatedness were controlled through the genomic relationship matrix. Association results were visualized using Manhattan plots with genome-wide significant and suggestive significance thresholds. λ and quantile–quantile (Q–Q) plots were used to assess the adequacy of population structure correction. Multiple testing correction was applied using the false discovery rate (FDR) method (Hochberg 1988). Accordingly, we assessed both the suggestive threshold and the genome-wide threshold (FDR correction) to detect outlier variants. In Manhattan plots, SNPs surpassing the genome-wide threshold were regarded as having a direct influence on phenotypic traits, whereas those falling between the suggestive and genome-wide thresholds were considered as potentially associated with phenotypic variation.

Significant and suggestive SNPs identified from the GWAS analyses were functionally annotated to identify candidate genes potentially associated with the analyzed traits. Gene annotation was performed using the *Ensembl* platform (https://www.ensembl.org/biomart/) based on the caprine reference genome assembly ARS1.2.

For SNPs located within protein-coding genes, direct gene annotation was conducted based on their genomic coordinates. For intergenic SNPs, candidate genes were identified within a 1 Mb flanking region (± 500 kb upstream and downstream) to capture potential regulatory variants that may influence gene expression in nearby loci. This window size was chosen based on LD decay patterns observed in these populations (Bilginer et al. 2025), where LD (r^2^) declined below 0.2 within approximately 200–500 kb across all breeds.

Additionally, the potential effects of identified candidate genes and genomic regions were validated by checking the results of previously reported studies in various livestock species.

## Results

This study was initially designed to conduct a GWAS analysis with 576 animals, while 95 samples were discarded from the experiment due to either failure in laboratory practices (DNA extraction and library preparation) or filtration criteria. The descriptive statistics for the studied phenotypic traits of the remaining animals are presented in Table 1.

**Table 1:** An overview of descriptive statistics of phenotypic traits per breed

Phenotypic trait	Breed	*n*	Gender	Mean (gr)	SD (gr)	Min (gr)	Max (gr)	CV (%)
BW	HAI	145	Female	3246,21	729,35	2000	5720	22,5
		11	Male	4059,09	594,38	3000	5300	14,6
		156	Total	3303,53	749,03	2000	5720	22,7
	HNM	161	Female	3711,96	599,80	2070	4980	16,2
		7	Male	3634,29	362,48	3000	4100	10,0
		168	Total	3708,75	591,29	2070	4980	15,9
	KBK	135	Female	3652,15	481,21	1900	5100	13,2
		22	Male	3806,36	622,52	2860	5300	16,4
		157	Total	3673,76	503,95	1900	5300	13,7
90-LW	HAI	145	Female	14584,14	2626,80	9600	20,800	18
		11	Male	22063,64	3628,78	15,500	28,400	16,4
		156	Total	15112,18	3309,33	9600	28,400	21,9
	HNM	161	Female	17469,13	3425,65	10,300	28,100	19,6
		7	Male	18914,28	2251,24	15,100	21,700	11,9
		168	Total	17529,35	3392,52	10,300	28,100	19,4
	KBK	135	Female	17259,48	2726,67	9600	26,100	15,8
		22	Male	27663,64	4169,35	21,700	35,800	15,1
		157	Total	18717,45	4674,81	9600	35,800	25,0
LMY	HAI	145	Female	94269,27	20099,58	43,050	156,750	21,3
	HNM	161	Female	106208,39	24603,32	42,000	177,150	23,2
	KBK	135	Female	102186,44	15088,48	70,500	133,650	14,8

*BW* birth weight, *90-LW* live weight at 90th day, *LMY* lactation milk yield, *HAI* hair, *HNM* Honamlı, *KBK* Kabakulak, *n* number of samples, *SD* standard deviation, *CV* coefficient of variation

Breed differences were assessed using Kruskal–Wallis test followed by Dunn’s post-hoc test with Bonferroni correction. For all traits, HAI differed significantly from HNM and KBK (*p* < 0.001), while HNM and KBK did not differ significantly (*p* > 0.05)

As illustrated in Table 1, HAI (3.3 and 15.1 kg) goats had lower values for BW and 90-LW compared to HNM (3.7 and 17.5 kg) and KBK (3.6 and 18.7 kg). Although KBK goats are born with a lower body weight (3.6 kg) than HNM goats (3.7 kg), they may reach a higher live weight at the 90th day (Table 1). HAI goats were of lower LMY value compared to HNM and KBK breeds (Table 1), which seems to be consistent with growth traits. Statistical comparisons revealed significant differences among breeds for all studied traits (Table 1). For BW, the Kruskal-Wallis test indicated significant variation among breeds (χ^2^ = 44.89, *p* < 0.001). Post-hoc analysis showed that HAI goats had significantly lower BW compared to both HNM (*p* < 0.001) and KBK (*p* < 0.001) goats, while no significant difference was observed between HNM and KBK (*p* = 1.000). Similarly, significant breed effects were detected for 90-LW (χ^2^ = 68.73, *p* < 0.001) and LMY (χ^2^ = 23.64, *p* < 0.001). For both traits, HAI goats showed significantly lower values than HNM (*p* < 0.001) and KBK (*p* < 0.001), whereas HNM and KBK did not differ significantly (90-LW: *p* = 0.261; LMY: *p* = 0.441). These results indicate that HAI goats are characterized by lower growth performance and milk production compared to HNM and KBK goats.

**Fig. 1:**
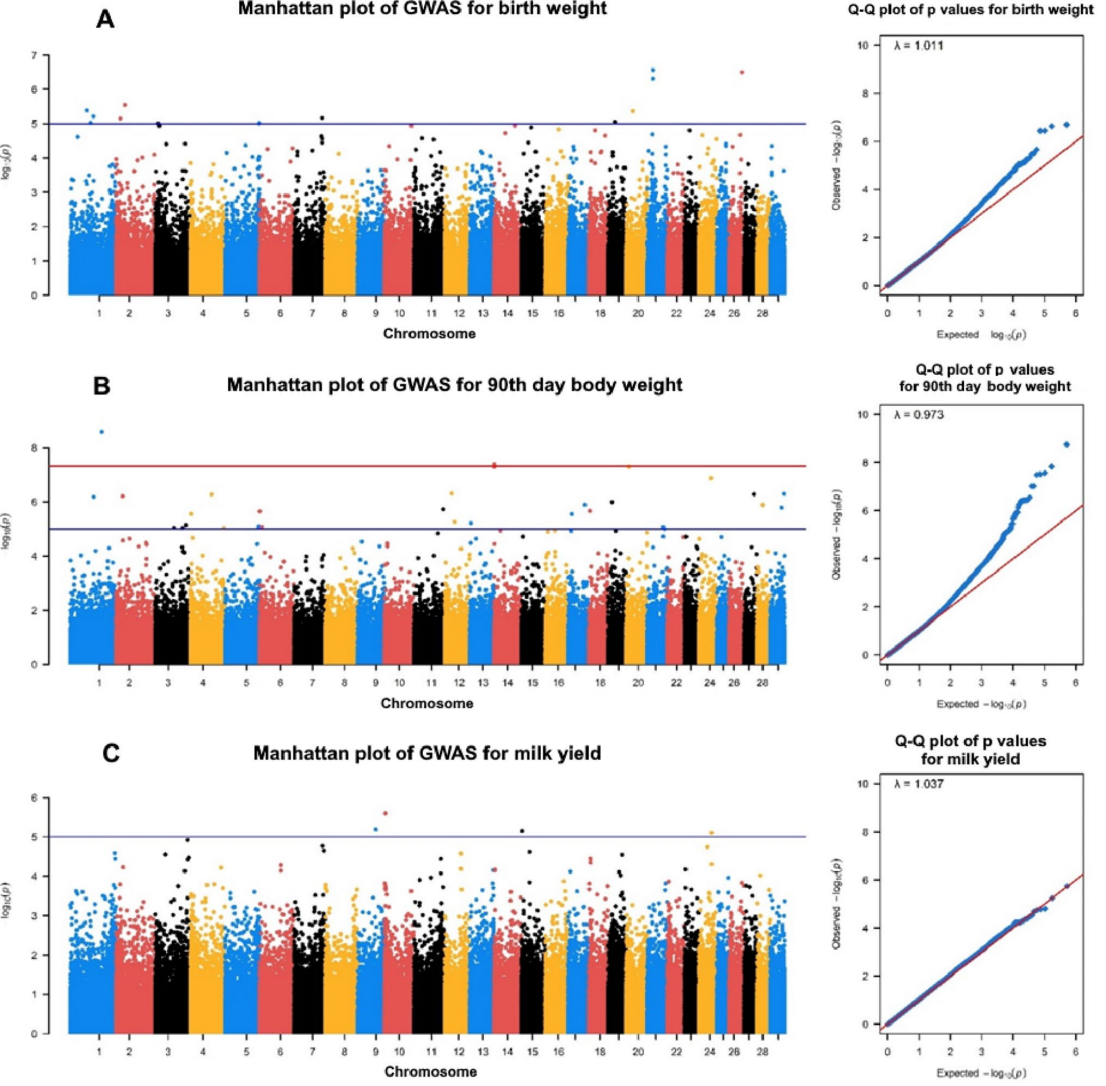
Manhattan and Q–Q plots for **A** BW, **B** 90-LW, and **C** LMY in HAI goats (blue and red lines in Manhattan plots represent to suggestive and genome-wide thresholds, respectively)

**Fig. 2:**
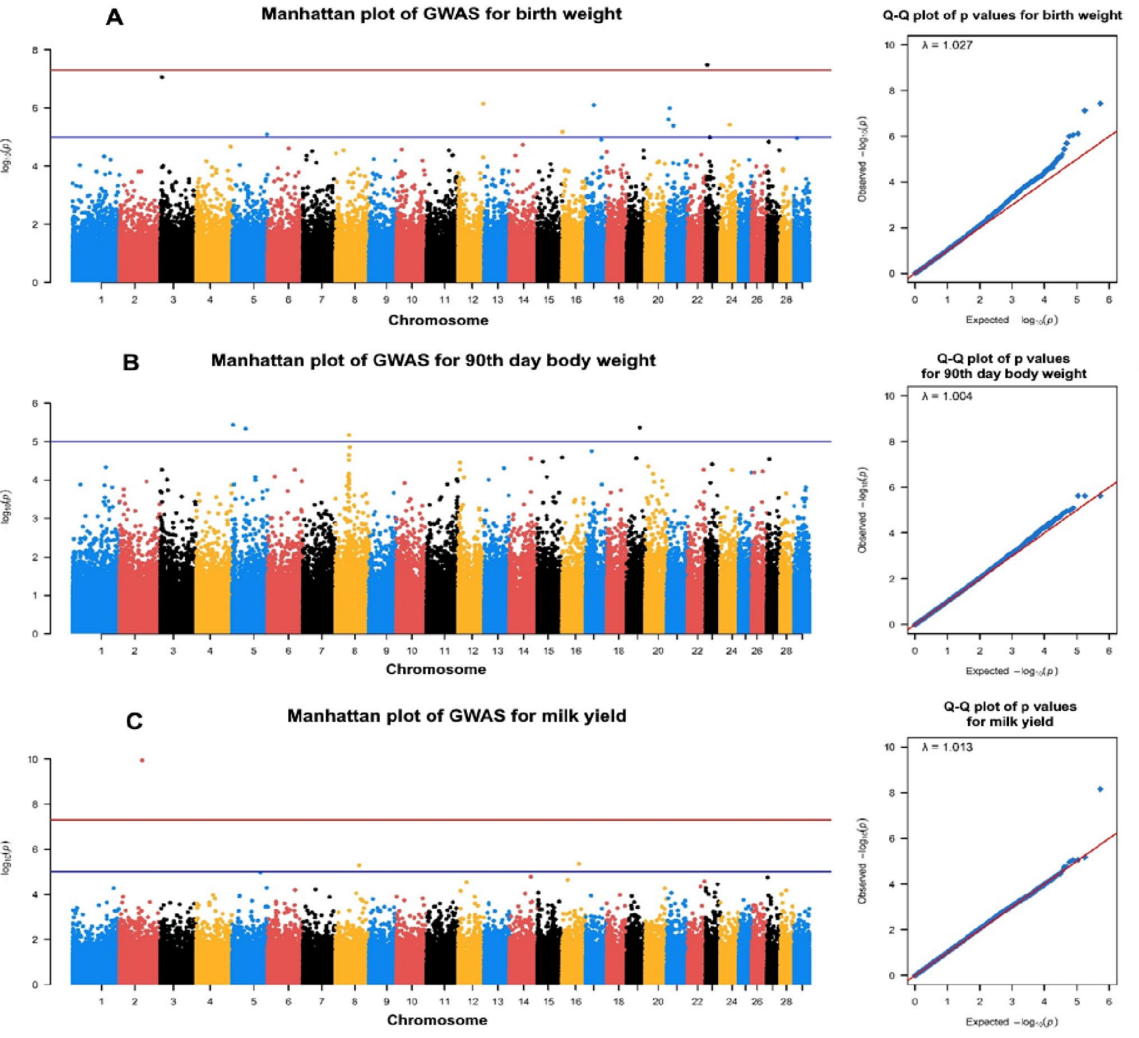
Manhattan and Q–Q plots for (A) BW, (B) 90-LW, and (C) LMY in HNM goats (blue and red lines in Manhattan plots represent to suggestive and genome-wide thresholds, respectively)

**Fig. 3:**
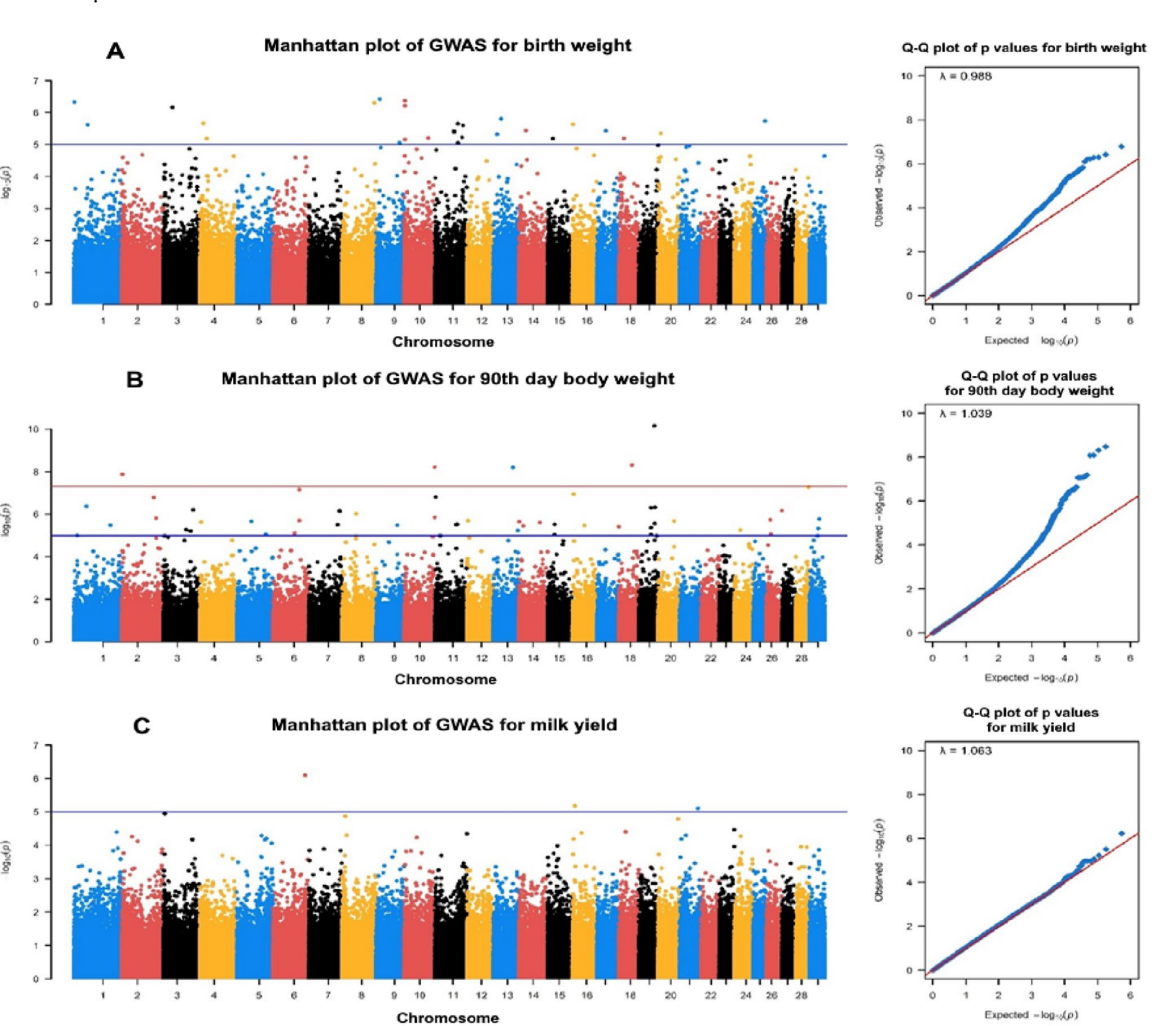
Manhattan and Q–Q plots for **A** BW, **B** 90-LW, and **C** LMY in KBK goats (blue and red lines in Manhattan plots represent to suggestive and genome-wide thresholds, respectively)

Manhattan and Q–Q plots of BW, 90-LW, and LMY for HAI, HNM, and KBK goats are given in Figs. 1 and 2, and 3, respectively. A total of 138 SNPs were outliers based on the suggestive threshold across all goats for the studied phenotypic traits. Annotation using the *Ensembl* database (https://www.ensembl.org/info/data/biomart/index.html) confirmed that 138 outlier SNPs were associated with 113 protein-coding genes, either directly overlapping (58 genes) or located nearby (55 genes). The lowest (20) and highest (71) numbers of outlier SNPs were identified in HNM and KBK, respectively, while this value was 47 SNPs for the HAI breed. At the trait level, the lowest (20) and highest (67) numbers of significant SNPs were identified for LMY and 90-LW, while 51 SNPs were detected for BW. The SNPs detected at the breed level were linked to 35, 17, and 61 genes in HAI, HNM, and KBK, respectively.

In the HAI breed, 16 of 47 SNPs overlapping 15 protein-coding genes (*PLCXD2*,* OSBPL11*,* LLP*,* AZIN2*,* SMARCAL1*,* CSMD2*,* UTP11*,* ZSWIM4*,* TBCA*,* ADCY8*,* ASIC2*,* RTN4RL1*,* IL6ST*,* ZNF10*, and *PRKG1*) turned out to be associated with BW (Table 2). Although a significant SNP was identified at chromosome 5, no protein-coding genes within a proximity of 500 kb upstream and downstream were detected. A total of 19 SNPs were detected to be associated with 90-LW in the HAI breed, in which 4 SNPs were identified within *NAMPT*,* RNFT2*,* ACACA*, and *AIFM2* genes, while other SNPs were at a range of 500 kb upstream and downstream of 12 protein-coding genes (Table 2). Similarly, 4 SNPs were observed to be related to LMY traits, which overlapped or were close to four protein-coding genes (*MAP7*,* KCNK10*,* SLC43A3*, and *PDE3B*) (Table 2).

In the HNM breed, a total of 20 SNPs exceeded the suggestive threshold, of which 12, 4, and 4 SNPs were associated with BW, 90-LW, and LMY traits. 5 SNPs of identified for BW directly overlapped with *DIAPH3*,* REN*,* GUCY1A3*,* MAK*, and *DLG2*, while remaining were located in the proximity of *IQCA1*,* TBC1D22A*,* ASB7*,* FURIN*,* ID*, and *SS18* genes (Table S1). Similarly, significant SNPs were detected within three protein-coding genes, most probably related to 90-LW (*APBA1*) and LMY (*GTSE1* and *SPATA17*) (Table S1).

Compared to other breeds, KBK contained a higher number of outlier SNPs, in which 23, 44, and 4 SNPs turned out to be associated with BW, 90-LW, and LMY. Most of the BW-associated SNPs directly overlapped with 12 protein-coding genes (*ADGRG7*,* STRA8*,* FOXN3*,* OXA1L*,* KLH29*,* TTLL11*,* PRKCQ*,* KIAA1217*,* PRR5L*,* HELZ*,* KICNIP1*, and *TMEM130*) (Table S1). Similarly, the numerous significant SNPs overlapped with 18 and 2 protein-coding genes identified for 90-LW (*IGSF21*,* DPYD*,* KCND3*,* PDE4DIP*,* SUGCT*,* SMC5*,* UTRN*,* MERTK*,* TPP2*,* DCDC2*,* PPP1R1B*,* SLC38A10*,* RNF213*,* RBFOX3*,* KIAA1328*,* PGBD5*,* KIRREL3*, and *OPCML*) and LMY (*COL6A3* and *WDR1*) traits, respectively (Tables S1, 2).

In this study, FDR correction was applied to increase the accuracy of identified genomic regions. In this regard, only 12 of 138 outlier SNPs exceeded the genome-wide significance threshold across the studied goat populations. As summarized in Table 2, among these genetic variants, 3 and 7 SNPs were directly overlapped with (*IGSF21*,* SLC38A10*, and *PGBD5*) or were located near some protein-coding genes (*ID4*,* MFSD1*,* CHMP4C*,* MAP1B*,* RALY*,* ZNF507*, and *CXCR4*). These SNPs were identified to be directly related to the studied phenotypic traits. Based on this criterion, two SNPs located at 12,380,234 position of chromosome 23 and at 75,366,873 position of 2, which overlapped with *ID4* and *CXCR4* genes, were detected in HNM. *ID4* and *CXCR4* genes were identified to significantly affect BW and LMY traits, respectively. However, no SNPs related to the 90-LW trait were observed in HNM goats. On the other hand, no SNPs associated with BW and LMY were identified in HAI and KBK goats (Table 2). In contrast, 8 genes were identified to be associated with 90-LW traits either in HAI (*MFSD1*, *CHMP4C*, and *MAP1B*) or KBK (*IGSF21*, *RALY*, *ZNF507*, *SLC38A10*, and *PGBD5*) goats (Table 2).

**Table 2:** Position and corresponding genes of SNPs exceeding the genome-wide threshold (FDR) in Anatolian goats

Trait	Breed	Chromosome	Position (bp)	Nearest gene	Distance (kb)	−log_10_(*p*)
BW	HAI	–	-	–	–	–
	HNM	23	12,380,234	*ID4*	111	7,05
	KBK	–	–	–	–	–
90-LW	HAI	1	108,225,908	*MFSD1*	38	8,20
		14	1,407,910	*CHMP4C*	5,3	7,03
		20	9,682,050	*MAP1B*	176	7,01
	HNM	–	–	–	–	–
	KBK	2	1,651,769	*IGSF21*	Within	7,89
		13	62,917,233	*RALY*	77	8,02
		18	43,790,753	*ZNF507*	229	8,04
		19	50,800,531	*SLC38A10*	Within	9,86
		28	42,830,428	*PGBD5*	Within	7,00
LMY	HAI	–	–	–	–	–
	HNM	2	75,366,873	*CXCR4*	386	7,20
	KBK	–	–	–	–	–

*BW* birth weight, *90-LW* live weight at 90th day, *LMY* lactation milk yield, *HAI* hair, *HNM* Honamlı, *KBK* Kabakulak, *bp* base pair, *kb* kilobase

A large part of the outlier SNPs (126), on the other hand, were between suggestive and significant thresholds (FDR correction), implying their potential effects on the studied phenotypic traits. Although their corresponding genes may represent variants with potential effects on the studied phenotypic traits, they should be interpreted with caution. Given the increased likelihood of false-positive signals at the suggestive level, these SNPs and their associated genes should be considered as hypothesis-generating rather than definitive targets for selection programs, and their relevance must be validated in independent and larger populations. Detailed information about these outlier SNPs, including their location and corresponding genomic regions, was given in Table S1.

## Discussion

The genetic architecture behind phenotypic traits in native Turkish livestock species has been mainly addressed by GWAS (Yilmaz et al. 2022; Erdoğan et al. 2024) and selection signatures analyses (Demir et al. 2023; Argun Karsli et al. 2024; Demir et al. 2025), which rely on SNP array and next-generation sequencing (NGS) technologies. However, no studies using NGS platforms such as ddRADseq are available in the literature to investigate the associations between genomic and phenotypic data in native Turkish goat breeds. Therefore, the results of the current study are promising for being a basis for designing further selection programs to improve growth and milk yield in local populations, since it was carried out with a moderate sample size (481 animals) and considerably high-density genetic data.

This study, employing an up-to-date statistical approach and molecular genotyping technique, identified a total of 138 outlier SNPs that overlapped with 58 and were located near 55 protein-coding genes, which are likely candidate genes affecting growth and milk traits in Anatolian goats. To confirm the effects of these candidate genes on phenotype, we further investigated the literature.

For example, *PLCXD2*,* LPP*,* TBCA*,* ADCY8*,* IL6ST*, and *CSMD2* genes were detected in the HAI breed for the BW trait. These genes were also reported to play a key role in the growth, muscling, body weight, conformation, and stature in various species and breeds (Lindholm-Perry et al. 2017; Sheet et al. 2021; Selionova et al. 2022; Ramos et al. 2023; Choudhury et al. 2023; Zhang et al. 2024). In HNM goats, 5 out of 12 SNPs associated with BW were located on or near the *IQCA1*, *DIAPH3*, *GUCY1A3*, *ASB7*, and *DLG2* genes. These genes have previously been reported to be associated with several phenotypic traits such as carcass weight, body conformation, body height, meat yield, and birth weight in various livestock species (Sheet et al. 2021; Tuersuntuoheti et al. 2023; Sood et al. 2023; Liu et al. 2023; Arikawa et al. 2024). Similarly, *TMEM130* and *FOXN3* identified for KBK goats were previously shown to affect body weight and related traits in livestock species (Wu et al. 2013; Deng et al. 2023).

Among the remaining candidate genes for Anatolian goats, *OSBPL11*, a member of the oxysterol-binding protein family, plays a role in lipid metabolism and has been reported to be involved in cholesterol and glucose metabolism in obese individuals (Bouchard et al. 2009; Jiang et al. 2024). The *AZIN2* gene promotes polyamine synthesis essential for cellular activity, including growth, differentiation, and apoptosis (Ramos-Molina et al. 2018; Mund et al. 2024). The interaction between *ZNF10* and the *TRIM28* gene may affect gene expression patterns, influencing cell proliferation and differentiation (Lorenz et al. 2022). Similarly, protein kinase is encoded by the *PRKG1* gene to regulate cell proliferation, differentiation, and growth. The *PRKG1* gene was reported to indirectly affect growth by controlling vascular smooth muscle relaxation and regulating blood pressure (Han et al. 2019).

The literature review also demonstrated that remaining protein-coding genes indirectly affect birth weight or take part in other biological pathways. For example, no studies have directly reported an association between the *SMARCAL1* gene and growth or growth-related pathways. However, it has been associated with feed intake in cattle (Abo-Ismail et al. 2018; Marín-Garzón et al. 2021), suggesting that *SMARCAL1* may indirectly influence growth. Besides, the *PRKG1* gene has been reported to be associated with various traits in livestock, including milk quality (Feng et al. 2024) and production (Ni et al. 2024), response to heat stress (Czech and Wang 2023), meat quality traits (Visser 2023), intramuscular fatty acid composition (Zeng et al. 2023), and tick resistance (Vajana et al. 2018). Similarly, the *CNTNAP2* gene, identified in this study as associated with birth weight, has previously been reported to be linked to milk production in various livestock species (George et al. 2023; Erdoğan et al. 2024), it is also known to play a significant role in cellular growth, differentiation, and immune function as well as growth in cattle (George et al. 2023). *FURIN* is required for trophoblast syncytialization by processing type 1 Insulin-like Growth Factor Receptor (*IGF1R*) (Zhou et al. 2014). The *FURIN* gene was reported to play a key role in fetal growth and birth weight in humans (Sabri 2013; Zhou et al. 2023), while it is responsible for carcass length in pigs (Falker-Gieske et al. 2019). ID1–ID4 genes encode multifunctional ID proteins that are widely expressed in almost all cells during embryonic development (Yokota and Mori 2002; Patel et al. 2015). These genes have been associated with growth traits, such as carcass length in pigs (Falker-Gieske et al. 2019), suggesting that their regulatory roles in cell proliferation and differentiation may influence body weight and growth in goats.

Regarding 90-LW, a total of 56 candidate genes were identified across HAI (16), HNM (3), and KBK (37) goats. Most of these genes were previously reported to be directly linked to growth traits. For example, among the candidate genes identified in the HAI breed, the *HRG* and *ACACA* were reported to be related to live weight in goats via GWAS analysis (Easa et al. 2022; Selionova et al. 2022). Similarly, several studies have reported associations between the *SHANK2* gene and backfat thickness in pigs (Ma et al. 2025), the *NAMPT* and *DOCK10* genes and intramuscular fat in pigs (Velez-Irizarry 2018; Li et al. 2022), the *SHH* gene and abdominal fat weight in chickens (Zhang et al. 2020), the *MAP1B* gene and BW in cattle (Campos et al. 2020), and the *RNFT2* gene and testicle thickness in roosters (Volkova et al. 2024).

In the HNM breed, three candidate genes were detected for 90-LW, in which *APBA1* and *PLEKHH3* have previously been reported to be associated with body weight and wither height (Saif et al. 2022; Krebs et al. 2023). Among the 44 SNPs identified as significant for 90-LW in KBK goats, 11 were located within or near the *AGTR1*, *DPYD*, *PDE4DIP*, *ILVBL*, *UTRN*, *GDF6*, *FAM84B*, *CDK18*, *RBFOX3*, *KIAA1328*, and *OPCML* genes. Literature review indicates that these genes have been reported to be associated with phenotypic traits such as carcass weight, muscle development, growth characteristics, skeletal development, intramuscular fat, body weight, fat deposition, and body length (Chang et al. 2018; Ding et al. 2019; Srikanth et al. 2020; Pan et al. 2021; Sheet et al. 2021; Atrian-Afiani et al. 2023; Deng et al. 2023; Long et al. 2024; Palacios Erazo et al. 2024).

The remaining candidate genes turned out to indirectly influence live weight and were associated with several biological pathways. To illustrate, the *CHMP4C* is involved in the endosomal sorting complex required for transport pathway (Casares-Arias et al. 2020), which plays a crucial role in cell division and signaling, potentially exerting an indirect influence on growth-related traits. The *TERF2IP* gene encodes a protein that plays a crucial role in telomere maintenance and transcription regulation (Deregowska and Wnuk 2021). This gene is involved in various metabolic pathways, notably regulating the NF-κB pathway, which regulates glycolysis, which is crucial for energy production (Mota et al. 2020). The integral endoplasmic reticulum proteins *VAPA* and *VAPB* participate in establishing ER contacts with multiple membranes by interacting with different tethers (Zhao et al. 2018). Therefore, lipid metabolism is needed for cellular energy balance, while mutations in the *VAPA* gene may negatively affect muscle and fat tissue development. The *AIFM2* gene is responsible for maintaining metabolism and cell survival, and controlling oxidative stress to support the growth and differentiation of muscle and fat cells (Peng et al. 2025). *IL26* gene, on the other hand, plays an important role in immune response (Poli et al. 2017) rather than growth traits. Similarly, the *TBK1* gene primarily functions in the regulation of innate immune responses, while it may influence growth due to its effects on cell proliferation and metabolism (Jia et al. 2020; Hui et al. 2025). No studies have directly linked the *KDR* gene to growth traits in livestock. However, this gene plays a critical role in embryonic development and tissue growth by regulating angiogenesis and vascular development through vascular endothelial growth factor signaling (Gogat et al. 2004; Ishitobi et al. 2011). The SMC5 protein, as part of the Smc5/6 complex, regulates DNA replication and chromosome segregation processes, thereby maintaining growth, particularly during cell division and organismal development (Peng and Zhao 2023; Lorite et al. 2024). High expression levels of *TPP2* have been reported to enhance cellular growth by accelerating the cell cycle and conferring resistance to apoptotic processes (Tomkinson 2019). SLC proteins play a critical role in cell metabolism and survival by regulating amino acid transport. Due to its similar functions, the *SLC38A10* gene particularly contributes to cell growth during developmental stages (Tripathi et al. 2021).

In this study, the lowest number of significant SNPs (20) and corresponding candidate genes (10) were identified for LMY. In the HAI breed, four SNPs associated with milk yield were located on or near the *MAP*, *KCNK10*, *SLC43A3*, and *PDE3B* genes. The *MAP* and *KCNK10* genes have previously been reported to be associated with lactation or milk production (Do et al. 2017; Sutera 2018). Although there is no direct evidence linking the *PDE3B* gene to milk yield, it has been identified as a candidate gene related to metabolic disorder ketosis in cattle (Klein et al. 2021). The *SLC43A3* gene has been reported in previous studies to be associated with traits such as fat deposition and reproduction (Rios et al. 2023; Cheng et al. 2024).

In the HNM breed, four SNPs associated with lactation milk yield were identified, of which one SNP was not located within or near any gene region. Of the genes associated with LMY, *CXCR4* and *SPATA17* have been previously reported to be linked to milk production (Abdel-Shafy et al. 2020; Bagnicka et al. 2023). Moreover, *CXCR4* is expressed in mammary gland tissues, implying its direct effects on milk traits as well as increasing the number of leukocytes during infections with *Escherichia coli* (Revskij et al. 2022). Four different SNPs associated with lactation milk yield were identified in the KBK population. Three of these SNPs are located in the *COL6A3*, *WDR1*, and *GSC* genes. Encoding the collagen type VI alpha 3 chain, the *COL6A3* gene is essential for maintaining the structural integrity of mammary glands, indirectly affecting epithelial cell differentiation and lactation capacity (Zanotti et al. 2023; Han et al. 2025). The *WDR1* regulates actin cytoskeleton remodeling in epithelial cells, which is essential for mammary alveolar development and milk secretion. Therefore, *WDR1* may indirectly affect milk production by influencing epithelial cell structure (Yuan et al. 2018; Tur-Gracia and Martinez-Quiles 2021). Although primarily known for its role in embryonic development and cell differentiation (Halder et al. 2014), the *GSC* gene may also indirectly affect milk production by regulating the development and differentiation of mammary gland epithelial cells.

This study revealed two key findings that significantly contribute to the literature. Firstly, the present study revealed that some SNPs associated with BW, 90-LW, and LMY have also been linked to different traits in the literature. It could be explained by the nature of quantitative inheritance and the diverse roles of protein-coding genes. Indeed, quantitative traits, including BW, 90-LW, and LMY, are influenced by multiple genes, while a single gene may participate in various biological pathways.

Secondly, KBK and HAI showed differences in terms of phenotypic records of studied traits and genetic analyses (number of significant SNPs and corresponding protein-coding genes). Previous studies based on molecular characterization also mentioned that KBK has become genetically different from the HAI (Karsli et al. 2020; Aktaş et al. 2024; Karslı and Demir 2024; Bilginer et al. 2025), while it is still considered a subvariety of the HAI breed. By integrating phenotypic and genomic data, this study demonstrated that KBK goats possess a higher number of significant SNPs and candidate genes than other breeds, particularly concerning the 90-LW trait. These findings suggest that the genomic differentiation of KBK goats from HAI goats may be attributed to several factors, including confined breeding practices and adaptation processes. Preserving the unique genomic variations identified in KBK goats is crucial for sustainable production and the conservation of genetic diversity.

## Conclusion

This study represents the first comprehensive GWAS analysis integrating ddRADseq-based high-density SNP data with phenotypic traits related to growth and milk yield in Anatolian goats, in which multiple candidate genes associated with BW, 90-LW, and LMY were identified. These findings offer a solid foundation for future genomic selection efforts to enhance the meat and milk yields of these native breeds via selective breeding based on identified genes. No common genes between the studied breeds were identified, indicating that specific selection programs should be designed for each goat breed to improve genetic gain regarding BW, 90-LW, and LMY traits. Another noteworthy finding of the study was the remarkably high number of candidate genes identified in KBK goats, a subvariety of HAI goats that is geographically restricted and has a relatively smaller population size compared to the other populations. This unexpected richness in genetic associations highlights the importance of conserving all subpopulations within a breed. Subgroups or varieties may harbor significant genetic diversity and possess unique genomic regions linked to adaptation and production traits, underscoring their potential value in future breeding and conservation programs. This study is still of some limitations, such as a comparatively low sample size per breed, imbalanced sex ratios within populations, a lack of other phenotypic records (fertility and resistance to disease and heat), and verification of changes in the expression profile of significant genes. Hence, further studies with diverse phenotypic records are recommended to address these gaps by focusing on a larger sample size and to screen the expression levels of significant genes, *ID4* and *CXCR4* genes for BW and LMY traits, respectively, in the HNM breed in particular.

## Supplementary Information

Below is the link to the electronic supplementary material.

Supplementary Material 1

## Data Availability

The data that support the findings of this study are available from the corresponding author upon reasonable request.
