# Supplementary material for: Candidate genes related to growth and milk production in three Anatolian goats revealed by GWAS
Source: Mamm Genome. 2026 Feb 2;37(1):30. doi: 10.1007/s00335-026-10203-w (PMC12864215; doi:10.1007/s00335-026-10203-w)
Supplement: Supplementary file 1 — Supplementary Material 1 [file 335_2026_10203_MOESM1_ESM.docx]

**Table S1.** Position and corresponding genes of SNPs between the suggestive and significant thresholds in Anatolian goats

| **Trait** | **Breed** | **Chromosome** | **Position (bp)** | **Nearest Gene** | **Distance (kb)** |
| --- | --- | --- | --- | --- | --- |
| **BW** | HAI | 1 | 56234643 | *PLCXD2* | within |
|  |  |  | 69817053 | *OSBPL11* |  |
|  |  |  | 78956818 | *LPP* | 55 |
|  |  | 2 | 15151361 | *AZIN2* | within |
|  |  |  | 31371981 | *SMARCAL1* |  |
|  |  | 3 | 9013185 | *CSMD2* | within |
|  |  |  | 12852412 | *UTP11* |  |
|  |  | 7 | 97371230 | *ZSWIM4* | 120 |
|  |  | 10 | 92379277 | *TBCA* | within |
|  |  | 14 | 72136042 | *ADCY8* |  |
|  |  | 19 | 15731549 | *ASIC2* | within |
|  |  |  | 22780987 | *RTN4RL1* |  |
|  |  | 20 | 23238000 | *IL6ST* |  |
|  |  | 21 | 20737324 | *ZNF10* |  |
|  |  | 26 | 43583365 | *PRKG1* |  |
|  | HNM | 3 | 4980540 | *IQCA1* | 7 |
|  |  | 5 | 116564335 | *TBC1D22A* | 433 |
|  |  | 12 | 83825079 | *DIAPH3* | within |
|  |  | 16 | 1320107 | *REN* |  |
|  |  | 17 | 27528464 | *GUCY1A3* |  |
|  |  | 21 | 5213395 | *ASB7* | 7 |
|  |  |  | 21085785 | *FURIN* | 1 |
|  |  | 23 | 4962165 | *MAK* | Within |
|  |  | 24 | 31104207 | *SS18* | 30 |
|  |  | 29 | 11355950 | *DLG2* | Within |
|  | KBK | 1 | 1086244 | *OLIG2* | 92 |
|  |  |  | 44862609 | *ADGRG7* | within |
|  |  | 4 | 11458933 | *CNTNAP2* | 161 |
|  |  |  | 21326614 | *STRA8* | within |
|  |  | 9 | 11309611 | *RNF146* | 11 |
|  |  |  | 77341900 | *RGS17* | 55 |
|  |  | 10 | 2620904 | *FOXN3* | within |
|  |  |  | 79393196 | *OXA1L* |  |
|  |  | 11 | 63166992 | *CEP68* | 7 |
|  |  |  | 75144561 | *KLHL29* | within |
|  |  |  | 92567315 | *TTLL11* |  |
|  |  | 13 | 11171912 | *PRKCQ* |  |
|  |  |  | 24411733 | *KIAA1217* |  |
|  |  | 14 | 25934601 | *EIF3E* | 5 |
|  |  | 15 | 16026332 | *PRR5L* | within |
|  |  | 16 | 761679 | *LAX1* | 115 |
|  |  | 17 | 28462446 | *PDGFC* | 143 |
|  |  | 18 | 17922476 | *CYLD* | 190 |
|  |  | 19 | 62337442 | *HELZ* | within |
|  |  | 20 | 2530047 | *KICNIP1* |  |
|  |  | 25 | 38295825 | *TMEM130* |  |
| **90-LW** | HAI | 1 | 80216155 | *HRG* | 1,3 |
|  |  | 2 | 22914496 | *DOCK10* | 22 |
|  |  | 4 | 2316861 | *SHH* | 71 |
|  |  |  | 72932780 | *NAMPT* | within |
|  |  | 11 | 100970589 | *PLPP7* | 9 |
|  |  | 17 | 12959124 | *RNFT2* | within |
|  |  |  | 58085367 | *SMAD1* | 73 |
|  |  | 18 | 4308342 | *TERF2IP* | 129 |
|  |  | 19 | 13134001 | *ACACA* | within |
|  |  | 24 | 42388676 | *VAPA* | 96 |
|  |  | 28 | 19313221 | *AIFM2* | within |
|  |  | 29 | 40466407 | *DAGLA* | 12 |
|  |  |  | 49221838 | *SHANK2* | 49 |
|  | HNM | 5 | 45086199 | *IL26* | 158 |
|  |  | 8 | 45438584 | *APBA1* | within |
|  |  | 19 | 42363493 | *PLEKHH3* | 10 |
|  | KBK | 1 | 40834331 | *ARL6* | 26 |
|  |  |  | 119336914 | *AGTR1* | 227 |
|  |  | 2 | 112947254 | *CDCA7* | 2 |
|  |  | 3 | 75025690 | *DPYD* | within |
|  |  |  | 89158596 | *KCND3* |  |
|  |  |  | 97503198 | *PDE4DIP* |  |
|  |  | 4 | 3996580 | *SUGCT* |  |
|  |  | 5 | 48575714 | *TBK1* | 39 |
|  |  |  | 49425871 | *TMEM5* | 150 |
|  |  | 6 | 71088718 | *KDR* | 26 |
|  |  |  | 85858186 | *LAGE33* | 17 |
|  |  | 7 | 93990211 | *TRAPPC5* | 82 |
|  |  |  | 100699711 | *ILVBL* | 6 |
|  |  | 8 | 46253461 | *SMC5* | within |
|  |  | 9 | 69121626 | *UTRN* |  |
|  |  | 11 | 3150550 | *MERTK* | within |
|  |  |  | 73038126 | *GAREM2* | 1 |
|  |  | 12 | 7369452 | *TPP2* | within |
|  |  | 14 | 1878948 | *SNX16* | 417 |
|  |  |  | 13991368 | *GDF6* | 147 |
|  |  |  | 68264857 | *FAM84B* | 141 |
|  |  | 16 | 2703531 | *CDK18* | 20 |
|  |  |  | 38842597 | *FASLG* | 173 |
|  |  | 18 | 244045 | *DCDC2* | within |
|  |  | 19 | 39656582 | *PPP1R1B* |  |
|  |  |  | 51500907 | *RNF213* |  |
|  |  |  | 52125485 | *RBFOX3* |  |
|  |  | 24 | 20451760 | *KIAA1328* | within |
|  |  | 26 | 13588200 | *FAM204A* | 183 |
|  |  | 29 | 8778042 | *EED* | 6 |
|  |  |  | 29447096 | *KIRREL3* | within |
|  |  |  | 32982238 | *OPCML* | within |
| **LMY** | HAI | 9 | 61443357 | *MAP7* | 27 |
|  |  | 10 | 3147320 | *KCNK10* | 47 |
|  |  | 15 | 2581832 | *SLC43A3* | 12 |
|  |  | 24 | 44412293 | *PDE3B* | within |
|  | HNM | 5 | 115644807 | *GTSE1* | Within |
|  |  | 8 | 80209696 | *-* | - |
|  |  | 16 | 19871696 | *SPATA17* | Within |
|  | KBK | 3 | 3930349 | *COL6A3* | within |
|  |  | 6 | 105608708 | *WDR1* | within |
|  |  | 21 | 58929082 | *GSC* | 4 |

**BW:** Birth weight; **90-LW:** Live weight at 90^th^ day; **LMY:** Lactation milk yield; **HAI:** Hair; **HNM:** Honamlı; **KBK:** Kabakulak; **bp:** Base pair; **kb:** kilobase.
